# Have immigrant children been left behind in COVID-19 testing rates? – A quantitative study in the Lisbon metropolitan area between march 2020 and may 2023

**DOI:** 10.3389/fpubh.2024.1286829

**Published:** 2024-03-12

**Authors:** Iolanda B. Alves, Silvia Panunzi, António C. Silva, Regina B. R. Loesch, Sofia C. R. Pereira, M. Rosário O. Martins

**Affiliations:** ^1^Global Health and Tropical Medicine (GHTM), Institute of Hygiene and Tropical Medicine (IHMT), NOVA University of Lisbon, Lisbon, Portugal; ^2^Unit of Epidemiology and Medical Statistics, Department of Diagnostics and Public Health, University of Verona, Verona, Italy; ^3^Public Health Department, Regional Health Administration of Lisbon and Tagus Valley, Ministry of Health, Lisbon, Portugal; ^4^AJPAS-Associação de Intervenção Comunitária, Desenvolvimento Social e de Saúde, Amadora, Portugal; ^5^Amadora Primary Care Health Centre’s Group, Regional Health Administration of Lisbon and Tagus Valley, Ministry of Health, Lisbon, Portugal

**Keywords:** access to health care, COVID-19 pandemic, testing for SARS-COV-2, COVID-19 testing rates, vulnerable population, immigrant children, social inequalities

## Abstract

Immigrant children often encounter additional barriers in accessing health care than their peers. However, there is a lack of evidence globally regarding how migrant status may have affected access to COVID-19 testing during the pandemic. This study aimed to analyze migrant status as a determinant of COVID-19 testing rates among children in the Lisbon metropolitan area, Portugal. This cross-sequential study included 722 children aged 2–8 years (47% non-immigrants; 53% immigrants). We collected data from a national surveillance system on laboratory-confirmed COVID-19 tests conducted between March 2020 and May 2023 and assessed whether children were ever tested for COVID-19 and testing frequency. We employed robust and standard Poisson regression models to estimate Adjusted Prevalence Ratios and Relative Risks with 95% confidence intervals. A total of 637 tests were performed. Immigrant children had lower testing rates (53% vs. 48%) and fewer tests per child (median: 2 vs. 3). Moreover, they were 17% less likely to be ever tested (PR = 0.83, 95% CI: 0.76–0.89) and performed 26% fewer tests (RR = 0.74, 95% CI: 0.67–0.82) compared to non-immigrant children. Caregiver’s age, education, employment status, child’s birth weight, and perceived health status were associated factors. Our findings suggest that the COVID-19 pandemic has left immigrant children somewhat behind. We conclude that specific interventions targeting vulnerable populations, such as immigrant children, are needed in future health crises.

## 1 Introduction

A complex interplay of factors, including cultural norms (e.g., beliefs about health practices), limited support networks (e.g., lack of family support), language barriers, and socioeconomic challenges, influence immigrants’ health-seeking behavior. Understanding these dynamics is crucial, particularly concerning children’s use of health services. Scientific evidence shows that immigrant children often have lower use of primary care and immunization services and higher use of emergency departments than their non-immigrant counterparts (1, 2).

The COVID-19 pandemic, comparable to other crises, had a disproportionately negative impact on the most vulnerable groups. Financial hardship, housing instability, and food shortages further exacerbated health inequalities between immigrants and non-immigrants (3–7).

During the pandemic, COVID-19 testing proved crucial to health care. Testing facilitated isolation, informed decision-making, and proper health care-seeking (8, 9). However, studies across different geographical locations reveal trends of delayed testing and variations in testing frequency for vulnerable populations despite their heightened susceptibility to infection and severe COVID-19 outcomes in contrast to the general population (10–12). In Italy, for example, compared with their non-immigrant counterparts, immigrants had an average delay of 2 weeks in taking the COVID-19 test (13). In North America, ethnic minority and immigrant populations, including children, showed reduced testing rates compared to the general population (14–16). Similarly, in Denmark, immigrants had lower testing rates than non-immigrants (10). In addition, two separate studies conducted in England highlighted the barriers faced by immigrant and minority children in accessing COVID-19 testing and treatment (12, 17).

Foreigners legally residing in Portugal can access free health care through the National Health Service (SNS). While most medical services are covered, some may have small fees (18). To ensure access to health care, including COVID-19 testing, immigrants with pending applications were granted temporary extensions to their stay between March and June 2020 (19). In addition, IOM Portugal produced multilingual brochures, DGS (Directorate General of Health) and ARS (Regional Health Administrations) to clarify rights and access to health care (20). However, inequalities in access to health care for immigrant children have been highlighted in a recent study. The study found that despite the higher use of primary health care services, immigrant children had fewer check-up visits at the age of four and used hospital emergency services more than non-migrant children before and during the pandemic (21). Still, there is a need for comprehensive data on access to COVID-19 testing among immigrants in Portugal.

In the aftermath of the pandemic, conducting a thorough, evidence-based analysis is essential to prepare for future public health crises. Understanding COVID-19 testing rates and the factors determining the testing frequency will help plan public health interventions to reduce health inequalities.

Our study aimed to estimate COVID-19 testing rates and their determinants among immigrant and non-immigrant children living in the Lisbon metropolitan area, Portugal.

## 2 Methods

### 2.1 Study design

The study employs a cross-sequential design, integrating key aspects of both cross-sectional and longitudinal methodologies to investigate COVID-19 testing rates and frequency in cohorts of children born in 2015, 2018, and 2020.

### 2.2 Study setting

**Figure 1:**
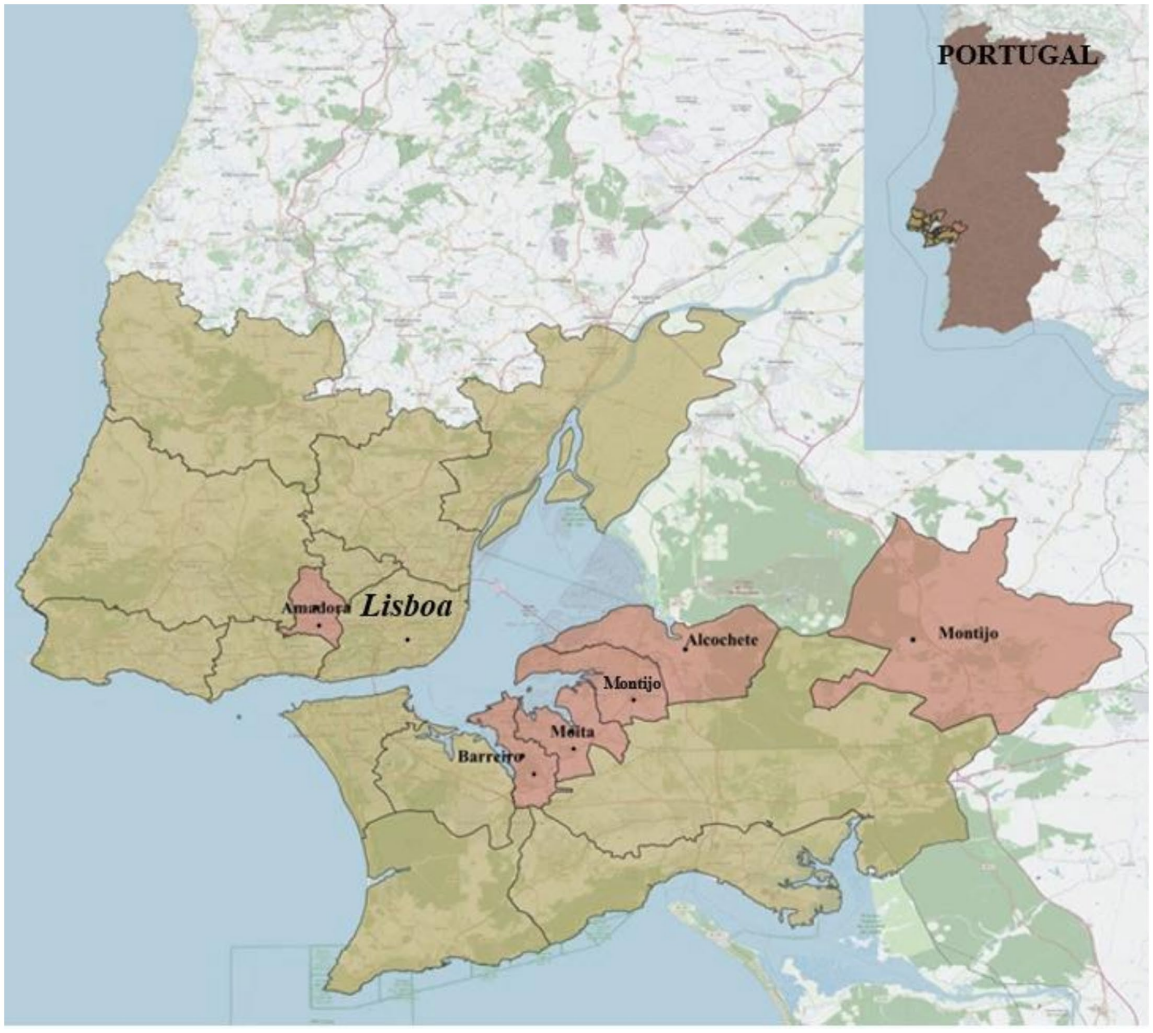
Lisbon metropolitan area, Portugal, highlighted in yellow, with the five municipalities under study marked in red. Created by the authors in QGIS Desktop (version 3.22.7).

The study was conducted in five municipalities in the Lisbon metropolitan area of Portugal, as presented in Figure 1. These municipalities collectively house a population of about 391 thousand, wherein approximately 68 thousand (17%) are foreign-born (22). These five municipalities comprise twenty-two primary health centers and three referral hospitals (23, 24).

### 2.3 Participants

The study included children aged 2 to 8, born in 2015, 2018, and 2020, living in five municipalities of the Lisbon metropolitan area, and attending primary health centers. Recruited children were categorized as immigrants if they resided in Portugal and were born outside the EU or had at least one parent born outside the EU. Primary health centers were randomly selected, and one non-immigrant child was matched to one immigrant in each center.

### 2.4 Recruitment

We recruited children based on their health and immunization appointment schedules at primary health care centers in their residence area. First, we selected children born in 2015, 2018, and 2020 from these schedules. Afterward, at the time scheduled, we approached all the parents/caregivers who attended a consultation or vaccination appointment with their child in the waiting room. Recruitment took place sequentially, with the enrollment of children born in 2015 starting in June 2019 (21). The enrollment of children born in 2018 and 2020 began in May 2022 and is ongoing.

### 2.5 Data collection

#### 2.5.1 Socioeconomic and demographic variables

At baseline (i.e., the first wave of data collection for each age cohort), we conducted face-to-face interviews with the child’s parents/caregivers using a questionnaire to collect socioeconomic and demographic data (Table 1). The interviews took place in a private area within the primary health care center to ensure complete privacy. Our research team included immigrant recruiters/interviewers with diverse backgrounds who have received specialized training to ensure accurate data collection. The interviews were conducted in Creole, Brazilian Portuguese, and English, in addition to Portuguese, as needed. The study examined various variables, including the caregiver’s age, sex, educational level, employment status, perception of the child’s health status, monthly household net income, family structure, child’s migrant status, birth weight, and health insurance.

**Table 1:** Characteristics of children tested and not tested for COVID-19 and their households.

Factors	No. (%) children					
	Overall		Not Tested		Tested	
Total	722	(100)	153	(100)	569	(100)
Predisposing factors						
Caregiver’s age (years); mean ± SD	34.85 ± 7.42		34.82 ± 7.75		34.86 ± 7.34	
Female caregiver	629	(87.1)	134	(87.6)	495	(87.0)
Caregiver’s educational level						
Less than secondary education	233	(32.3)	52	(34.0)	181	(31.9)
Upper secondary education	287	(39.8)	65	(42.5)	222	(39.1)
University degree/professional education	201	(27.9)	36	(23.5)	165	(29.0)
Caregiver’s employment status						
Employed	522	(72.9)	105	(69.5)	417	(73.8)
Family structure						
Single parent family	437	(60.6)	89	(58.6)	348	(61.2)
Household density^b^						
High density	615	(85.3)	129	(84.3)	486	(85.6)
Child’s migrant status						
Non-immigrant	340	(47.1)	42	(27.5)	298	(52.4)
Immigrant	382	(52.9)	111	(72.6)	271	(47.6)
Enabling factors						
Household net income/month						
> 750€^a^	503	(73.3)	107	(75.4)	396	(72.8)
Child’s health insurance						
Have	308	(43.0)	53	(35.1)	255	(45.1)
Need factors						
Child’s birth weight						
High (≥ 4 kg)	46	(6.7)	10	(6.8)	36	(6.6)
Normal (≥ 2.5 - < 4 kg)	568	(82.3)	114	(77.6)	454	(83.6)
Low (≥ 1.5 - < 2.5 kg)/Very low (< 1.5 kg)	76	(11.0)	23	(15.6)	53	(9.8)
Child’s perceived health status						
Very good	228	(31.8)	49	(32.5)	179	(31.6)
Good	344	(48.0)	75	(49.7)	269	(47.5)
Reasonable	128	(17.9)	26	(17.2)	102	(18.0)
Bad/Very bad	17	(2.4)	1	(0.7)	16	(2.8)

a In Portugal, since January 1, 2023, the minimum gross income is €760.

b Household density was defined as the ratio of people to the number of bedrooms and high HD as a ratio > 1.

#### 2.5.2 Notifications of laboratory-confirmed COVID-19 cases

The laboratory-confirmed cases of COVID-19, identified through RT-PCR and antigen tests, were obtained from Portugal’s General Directorate of Health’s laboratory surveillance system for COVID-19 (SINAVE Lab). These test results were categorized into two primary outcomes. Data collection spanned from March 31, 2020, to May 18, 2023, aligning with the World Health Organization Director’s declaration of COVID-19 ending as a public health emergency.

#### 2.5.3 Linkage

Individual data from the questionnaires were linked to laboratory-confirmed COVID-19 cases using a unique identification number; all relevant variables were then compiled in an Excel file.

### 2.6 Main outcomes

We analyzed two primary outcomes: the binary variable “ever tested for COVID-19,” indicating whether the child had performed at least one COVID-19 test during the study period (yes/no), and “COVID-19 testing frequency” as the sum of all COVID-19 tests performed during the study period.

### 2.7 Independent variables

We based our selection of independent variables on Andersen and Newman’s model in its revisited version (25). This approach was also used to assess factors associated with COVID-19 testing among adults and to evaluate health care utilization in European countries (26). Andersen and Newman’s model outlines three main categories of factors that collectively impact individuals’ utilization of health care services: 1 - predisposing factors, 2 - enabling factors, and 3 - perceived need to use health care factors. Accordingly, we included the following variables in the study: caregiver’s age and sex (male/female), family structure (single parent family/traditional family), child migrant status (non-immigrant/immigrant), household density (HD) (high/low), caregiver’s education (less than secondary/upper secondary/university degree), caregiver’s employment status (unemployed/employed). In this study, we defined HD as the ratio of people to the number of bedrooms in the household, and we considered a high HD as a ratio greater than 1. In addition, we included monthly net household income (≤750€/>750€) and whether the child has health insurance (have/do not have), the child’s birth weight (high/normal/low/very low), and the caregiver’s perception of the child’s health status (very good/good/reasonable/poor and very poor). We included children’s birth weight as a variable for perceived health care needs because it significantly influences the likelihood of mortality, morbidity, and disability during the critical early stages of life, particularly for newborns, infants, and children (27).

### 2.8 Statistical analysis

We calculated the percentage of children ever tested for COVID-19 with a 95% confidence interval, computed the median and a separate interquartile range for the number of tests performed, and further disaggregated both measures by migrant status. We used t-tests or non-parametric Mann–Whitney U tests to compare quantitative variables between groups, depending on their distribution; adjusted a Poisson regression, a log-linear model used for count data, with robust variance to estimate the determinants of ever tested for COVID-19 (28); adjusted a standard Poisson regression to estimate the determinants of COVID-19 testing frequency, and calculated unadjusted and adjusted prevalence ratios (aPR) and risk ratios for both models with a 95% confidence interval (Tables 2, 3). Furthermore, we estimated VIF coefficients from the adjusted models to investigate potential multicollinearity between independent variables and the two primary outcomes. We considered the general rule of thumb: VIFs >5 warrant further investigation of possible multicollinearity (Table 4).

**Table 2:** Bivariate analysis of determinants associated with ever tested and testing frequency for COVID-19.

Factors	Children tested v. not tested			Total no. of tests performed		
	uPR	95% CI	*p* - value	uRR	95% CI	*p* - value
Predisposing factors						
Caregiver’s age (years)	1.00	[0.99–1.01]	0.977	1.00	[0.99–1.01]	0.691
Female caregiver (Ref. = male)	0.99	[0.88–1.11]	0.929	1.22	[0.78–1.27]	0.006
Caregiver’s education (Ref. = less than secondary)						
Upper secondary education	1.00	[0.91–1.09]	0.966	1.01	[0.82–1.21]	0.898
University degree/professional	1.06	[0.96–1.16]	0.608	1.13	[0.86–1.30]	0.040
Caregiver’s employment status (Ref. = unemployed/student/retired)						
Employed	1.05	[0.96–1.15]	0.630	1.21	[0.87–1.27]	<0.001
Family structure (Ref. = traditional family)						
Single parent family	1.02	[0.95–1.11]	0.788	1.03	[0.87–1.21]	0.464
Household density^b^ (Ref. = low density)						
High density	1.02	[0.91–1.14]	0.858	1.01	[0.81–1.3]	0.932
Child’s migrant status (Ref. = non-immigrant)						
Immigrant	0.81	[0.75–0.87]	0.012	0.69	[0.69–0.95]	<0.001
Enabling factors						
Household net income/month^a^ (Ref. = ≤ 750€)						
>750€	0.97	[0.89–1.06]	0.780	1.00	[0.81–1.18]	0.982
Child’s health insurance (Ref. = not have)						
Have	1.09	[1.01–1.17]	0.310	1.21	[0.92–1.29]	<0.001
Need factors						
Child’s birth weight (Ref. = low (< 2.5 kg))						
Normal (≥ 2.5 - < 4 kg)	1.15	[0.98–1.34]	0.347	1.24	[0.87–1.54]	0.007
High (≥ 4 kg)	1.12	[0.90–1.39]	0.593	0.99	[0.73–1.71]	0.944
Child’s perceived health status (Ref. = very good)						
Good	1.00	[0.91–1.09]	0.967	1.16	[0.83–1.20]	0.005
Reasonable	1.02	[0.91–1.13]	0.904	1.24	[0.79–1.29]	0.001
Bad/Very bad	1.20	[1.04–1.38]	0.487	1.44	[0.69–1.93]	0.009

a In Portugal, since January 1, 2023, the minimum gross income is €760.

b Household density was defined as the ratio of people to the number of bedrooms and high HD as a ratio > 1.

CI, confidence interval; uPR, unadjusted prevalence ratio; uRR, unadjusted rate ratio; Ref., reference category.

**Table 3:** Multivariate analysis of determinants associated with ever tested for COVID-19 and testing frequency.

Factors	Children tested v. not tested			Total no. of tests performed		
	aPR	95% CI	*p*-value	aRR	95% CI	*p*-value
Predisposing factors						
Caregiver’s age (years)	1	[0.99–1.01]	0.76	0.99	[0.99–1.00]	0.05
Female caregiver (Ref. = male)	0.98	[0.90–1.06]	0.55	0.95	[0.86–1.04]	0.27
Caregiver’s education (Ref. = less than secondary)						
Upper secondary education	1.01	[0.91–1.12]	0.83	1.02	[0.91–1.15]	0.73
University degree/professional	1.06	[0.95–1.18]	0.33	1.14	[1.00–1.30]	0.05
Caregiver’s employment status (Ref. = unemployed/student/retired)						
Employed	0.99	[0.90–1.09]	0.87	1.16	[1.03–1.31]	0.01
Family structure (Ref. = traditional family)						
Single parent family	0.97	[0.89–1.06]	0.53	0.98	[0.88–1.09]	0.67
Household density^b^ (Ref. = low density)						
High density	1.09	[0.96–1.22]	0.17	1.12	[0.98–1.29]	0.11
Child’s migrant status (Ref. = non-immigrant)						
Immigrant	0.83	[0.76–0.89]	< 0.001	0.74	[0.67–0.82]	< 0.001
Enabling factors						
Household net income/month (Ref. = ≤ 750€)						
> 750€^a^	0.97	[0.89–1.06]	0.46	0.94	[0.84–1.06]	0.31
Child’s health insurance (Ref. = not have)						
Have	1.03	[0.95–1.12]	0.45	1.10	[0.99–1.23]	0.067
Need factors						
Child’s birth weight (Ref. = low (< 2.5 kg))						
Normal (≥ 2.5 - < 4 kg)	1.16	[0.98–1.36]	0.08	1.19	[1.01–1.41]	0.04
High (≥ 4 kg)	1.16	[0.94–1.44]	0.17	1.02	[0.79–1.31]	0.87
Child’s perceived health status (Ref. = very good)						
Good	1.00	[0.92–1.10]	0.95	1.14	[1.02–1.27]	0.023
Reasonable	1.02	[0.91–1.14]	0.76	1.26	[1.10–1.45]	0.001
Bad/Very bad	1.15	[1.00–1.32]	0.05	1.36	[1.02–1.79]	0.032

a In Portugal, since January 1, 2023, the minimum gross income is €760.

b Household density was defined as the ratio of people to the number of bedrooms and high HD as a ratio > 1.

CI, confidence interval; aPR, adjusted prevalence ratio; aRR, adjusted rate ratio; Ref., reference category; Models are fully adjusted for all the independent variables listed in the table.

**Table 4:** Multicollinearity analysis for determinants associated with ever tested and testing frequency for COVID-19.

Factors	Children tested v. not tested			Total no. of tests performed		
	GVIF	Df	$\sqrt[{2}]{\frac{\mathrm{GVIF}}{Df}}$	GVIF	Df	$\sqrt[{2}]{\frac{\mathrm{GVIF}}{Df}}$
Caregiver’s age	1.10	1.00	1.05	1.11	1.00	1.05
Caregiver’s sex	1.07	1.00	1.03	1.06	1.00	1.03
Caregiver’s education	1.24	2.00	1.05	1.25	2.00	1.06
Caregiver’s employment status	1.13	1.00	1.06	1.12	1.00	1.06
Family structure	1.19	1.00	1.09	1.19	1.00	1.09
Household density	1.09	1.00	1.05	1.09	1.00	1.04
Child’s migrant status	1.19	1.00	1.03	1.07	1.00	1.04
Household net income/month	1.13	1.00	1.06	1.11	1.00	1.05
Child’s health insurance	1.25	1.00	1.12	1.25	1.00	1.12
Child’s birth weight	1.04	2.00	1.01	1.04	2.00	1.01
Child’s perceived health status	1.08	3.00	1.01	1.08	3.00	1.01

GVIF, Generalized variance inflation factor; Df, Degrees of freedom.

We set our significance level at 5% and performed the statistical analysis in R software (version 4.0.3) using the packages sandwich, MASS, car, and dplyr. We generated figures using Excel (Microsoft Professional Plus 2019).

### 2.9 Ethical approval

The Ethics Committee for Health of the Regional Health Administration of Lisbon and Tagus Valley, Portugal (001/CES/INV/2019 and 071/CES/INV/2021, respectively) approved both cohort studies and COVID-19 interim study (9-2020/CES/2020). Moreover, one of the child’s primary caregivers signed an information and consent form before participating in the study.

## 3 Results

Of the parents/caregivers approached, 32 (4.2%) declined to participate. The main reasons for rejection were lack of interest, not being the primary caregiver, and time constraints. We enrolled a total of 722 children across three age groups: 420 in age group 1 (born in 2015), 133 in age group 2 (born in 2018), and 169 in age group 3 (born in 2020). From March 2020 to May 2023, the participants completed 637 tests. The number of tests per child ranged from 0 to 12, with a median of 2. Out of all participants, 78.8% (95% CI: 75.8–81.7) underwent at least one COVID-19 test; 52.4% (298) were non-immigrants, and 47.6% (271) were immigrants (*p* < 0.001). The non-immigrant children underwent a median of 3 COVID-19 tests (range: 1.75–5.00), whereas immigrant children underwent a median of 2 tests (range: 0.00–3.00), with a statistically significant difference (p < 0.001). Most immigrants were from three Portuguese-speaking African countries and Brazil, among which Cape Verde accounted for 25.9%, Angola for 15.7%, Brazil for 14.7%, Guinea Bissau for 12.6%, São Tomé and Principe for 7.3%, and Mozambique for 2.9%. Additionally, we enrolled children from Africa, Asia, South America, North America, Northern Europe, and Eastern Europe. Table 1 provides further information on children’s socioeconomic and demographic characteristics.

### 3.1 Determinants of ever tested for COVID-19

**Figure 2:**
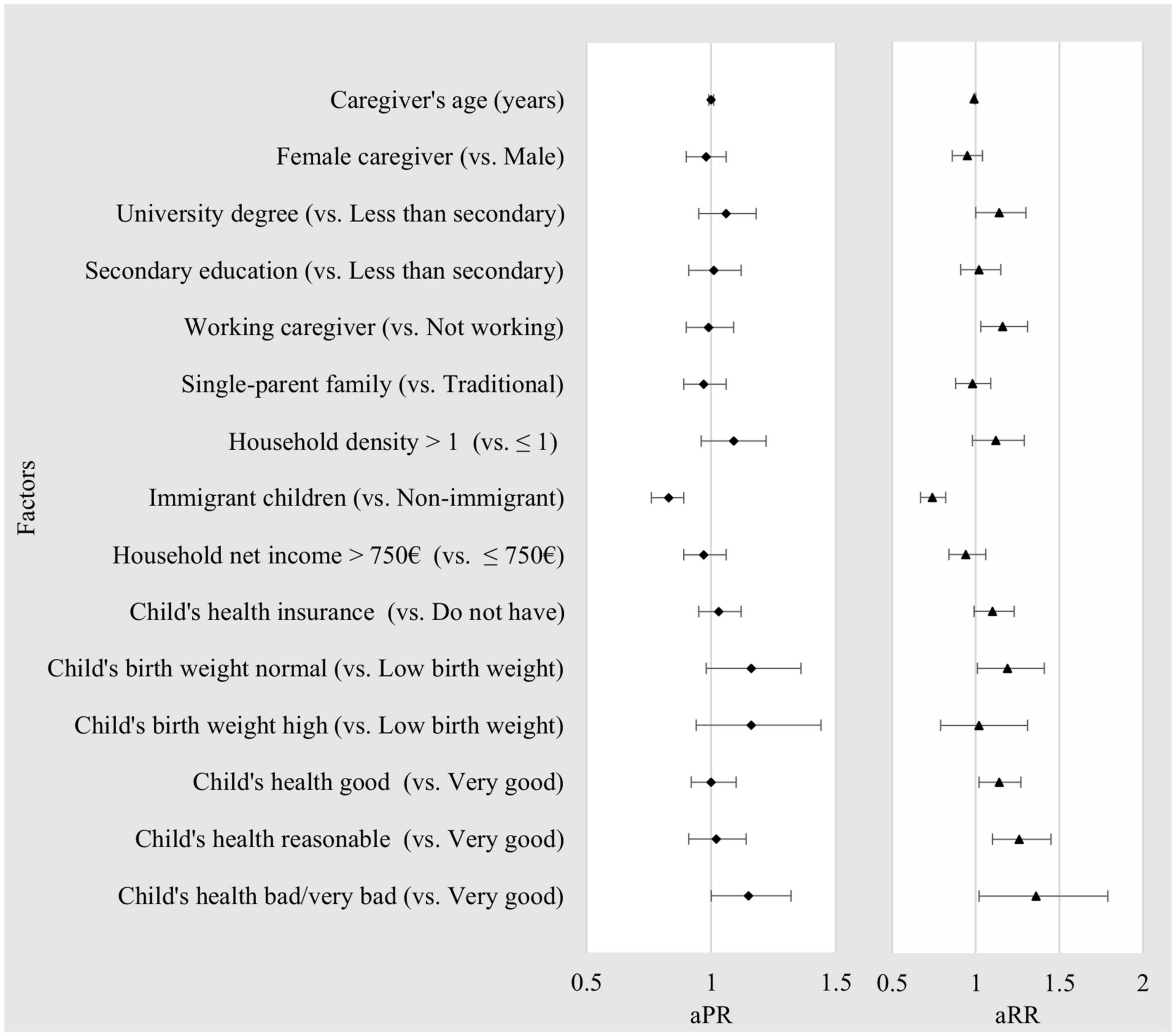
Factors influencing COVID-19 testing rates and testing frequency in children. aPR, Adjusted prevalence ratio; aRR, Adjusted rate ratio; Models were fully adjusted for all the independent variables listed.

After adjusting for other variables, non-immigrant children were 17% more likely to be ever tested for COVID-19 than immigrant children (PR = 0.83, 95% CI: 0.76–0.89, *p* < 0.001). Furthermore, children with caregivers who considered their health to be poor or very poor were 1.15 times more likely to be ever tested for COVID-19 than those with caregivers who rated their health as excellent (PR = 1.15, 95% CI: 1.00–1.32, *p* < 0.05). Table 3 and Figure 2 display additional determinants linked to predisposing, enabling, and health care necessity factors.

### 3.2 Determinants of COVID-19 testing frequency

As shown in Table 3 and Figure 2, immigrant children underwent 26% fewer COVID-19 tests than non-immigrant children when adjusting for other variables (rate ratio (RR) = 0.74, 95% CI: 0.67–0.82, *p* < 0.001). Also, children whose parents/caregivers had university degrees or professional education underwent 1.14 times more tests than caregivers with less than secondary education (RR = 1.14, 95% CI: 1.00–1.30, *p* < 0.05). Children with older caregivers were associated with less frequent testing. (RR = 0.99, 95% CI: 0.99–1.00, *p* < 0.05). Children whose caregivers were employed had a 1.16 times greater likelihood of being tested than those with caregivers who were unemployed, students, or retired (RR = 1.16, 95% CI: 1.03–1.31, *p* < 0.01). Moreover, children with an average birth weight underwent more tests than those with low birth weight (RR = 1.19, 95% CI: 1.01–1.41, *p* < 0.04). Children whose caregivers rated their health as good, fair, or bad had higher testing rates than those with very good health (RR = 1.14, 95% CI: 1.02–1.27; RR = 1.26, 95% CI: 1.10–1.45; RR = 1.36, 95% CI: 1.02–1.79, respectively).

## 4 Discussion

Our study fills a critical research gap by presenting data on access to health care among immigrant children in Portugal, focusing on COVID-19 testing during the pandemic. The primary objective was to examine immigration as a determinant of COVID-19 testing among children residing in the Lisbon metropolitan area, Portugal. First, we compared testing rates between immigrants and non-immigrants and calculated the median number of tests performed; second, we identified factors influencing testing patterns. Although some international studies have explored COVID-19 testing rates among immigrants, most have concentrated on other at-risk populations (29–34).

We found that a significant number of children (78.8%) underwent COVID-19 testing between March 2020 and May 2023, indicating, indicating extensive testing coverage for the pediatric population in the Lisbon region. However, we also observed a significant disparity in testing rates between immigrant and non-immigrant children. Our findings align with previous research on various vulnerable groups in North America. For example, two studies conducted in North Carolina revealed that ethnic minorities completed 18 to 31% and 1.4 to 7.5% fewer tests than the general population (14, 30). Similarly, individuals with greater social vulnerability performed 37 to 48% fewer tests in San Francisco, USA (31).

In addition, our results indicated that immigrant children were 17% less likely to undergo COVID-19 testing than their non-immigrant counterparts after adjusting for other variables. Consistent with this, a study conducted in North Carolina found that ethnic minorities were 7 to 33% less likely than the general population to undergo testing without adjusting for other variables (30). Furthermore, neighborhoods with residents of low income and communities at higher risk of COVID-19 were less likely to undergo testing in Toronto (32).

Moreover, we found that immigrant children underwent 26% fewer COVID-19 tests than non-immigrants. These findings suggest that immigrant children residing in the Lisbon metropolitan area experience limited access to COVID-19 testing compared to their peers, as observed in a study with adult immigrants conducted in Italy (29).

Our study on determinants of testing frequency found that factors beyond migrant status were significant in explaining the likelihood of testing. Specifically, after adjusting for other variables, the probability of favorable outcomes for children is higher when their caregivers possess a university degree or professional education (rather than less than secondary education), when they have an older parent or caregiver (as opposed to younger ones) when their caregivers are employed (as opposed to unemployed, students, or retired), when they have an average birth weight (as opposed to low birth weight), and when their caregivers perceive their health as good, fair, or poor (versus very good perceived health). Various barriers to health care outlined in existing literature may contribute to disparities in access to COVID-19 testing between immigrant and non-immigrant children (2).

These disparities are likely rooted in social determinants, such as living in areas with restricted access to public transportation services and limited schedules, lacking health insurance or being concerned about associated costs, facing occupational constraints like the inability to take time off work or having no paid leave, and harboring distrust toward government and healthcare systems (9).

In 2022, 16.7% of children born in Portugal had mothers of foreign origin, emphasizing the critical influence of immigration on developing the country’s health policies (35). Given the significance of this population group, our research findings have important implications for effectively managing public health crises in Portugal.

Children infected with COVID-19 who have not been tested pose a risk by potentially not following isolation guidelines and facilitating virus transmission, which is especially concerning in densely populated areas and households with multiple generations (4, 36, 37). On the other hand, if immigrant children have limited access to testing, the reported COVID-19 positivity rates may be underestimated. This is a significant concern since this population is already overrepresented in global cases (10). In addition, it is crucial to note that COVID-19 testing is vital for epidemiological surveillance, giving essential information for decision-making regarding mitigation measures and the efficient allocation of public health resources (38).

We conducted this study solely within the confines of the Lisbon metropolitan area, and the results cannot be generalized to other regions of Portugal. Nevertheless, it is important to stress that we commenced this cohort study before the pandemic and focused on variables and outcomes unrelated to COVID-19.

Further studies, including qualitative research, can improve our comprehension of the barriers and hesitation experienced by immigrant families when accessing COVID-19 testing. Such insights supplement and reinforce the results of our study.

In conclusion, our findings have significant ramifications, particularly in forthcoming public health crises. We have clarified the influence of migrant status as a noteworthy risk factor in COVID-19 testing for children. Furthermore, our study revealed additional significant factors contributing to the variation in testing frequency among pediatric populations. These findings emphasize the need for targeted interventions to reduce health care disparities among children in the Lisbon metropolitan area.

## Data availability statement

The raw data supporting the conclusions of this article will be made available by the authors, without undue reservation.

## Author contributions

IA: Conceptualization, Data curation, Formal analysis, Investigation, Methodology, Validation, Writing – original draft, Writing – review & editing. SiP: Formal analysis, Investigation, Methodology, Writing – original draft, Writing – review & editing. AS: Conceptualization, Writing – review & editing. RL: Investigation, Writing – review & editing. SoP: Investigation, Writing – review & editing. MM: Conceptualization, Data curation, Formal analysis, Funding acquisition, Investigation, Methodology, Project administration, Supervision, Validation, Writing – original draft, Writing – review & editing.
